# Improvement of Anomalous Behavior Detection of GNSS Signal Based on TDNN for Augmentation Systems

**DOI:** 10.3390/s18113800

**Published:** 2018-11-06

**Authors:** Daehee Kim, Jeongho Cho

**Affiliations:** 1 Department of Internet of Things, Soonchunhyang University, Asan 31538, Korea; daeheekim@sch.ac.kr; 2 Department of Electrical Engineering, Soonchunhyang University, Asan 31538, Korea

**Keywords:** global navigation satellite system, receiver autonomous integrity monitor, time delay neural network, integrity monitoring, augmentation systems

## Abstract

The reliability of a navigation system is crucial for navigation purposes, especially in areas where stringent performance is required, such as civil aviation or intelligent transportation systems (ITSs). Therefore, integrity monitoring is an inseparable part of safety-critical navigation applications. The receiver autonomous integrity monitor (RAIM) has been used with the global navigation satellite system (GNSS) to provide integrity monitoring within avionics itself, such as in civil aviation for lateral navigation (LNAV) or the non-precision approach (NPA). However, standard RAIM may not meet the stricter aviation availability and integrity requirements for certain operations, e.g., precision approach flight phases, and also is not sufficient for on-ground vehicle integrity monitoring of several specific ITS applications. One possible way to more clearly distinguish anomalies in observed GNSS signals is to take advantage of time-delayed neural networks (TDNNs) to estimate useful information about the faulty characteristics, rather than simply using RAIM alone. Based on the performance evaluation, it was determined that this method can reliably detect flaws in navigation satellites significantly faster than RAIM alone, and it was confirmed that TDNN-based integrity monitoring using RAIM is an encouraging alternative to improve the integrity assurance level of RAIM in terms of GNSS anomaly detection.

## 1. Introduction

In areas that require stringent performance when using the global navigation satellite system (GNSS), such as civil aviation or intelligent transportation systems (ITSs) (e.g., electronic fee collection, route guidance, advanced driver assistance systems including collision avoidance systems, and intelligent speed adaptation), the integrity of GNSS plays an important role and its monitoring is required. In these liability-critical applications, the reliability of location solutions derived from navigation data must be considered [1,2,3,4]. This measure of reliability is referred to as the integrity of the navigation system and is a feature that notifies users in time when it is undesirable to use a navigation system. Unacceptable position deviations can have a significant impact on positioning and navigation functions, significantly degrading performance; thus, integrity monitors need to be utilized for the detection and exclusion of abnormal signal sources before estimating a user’s position [1,2,5].

GNSS provides some basic integrity information to users, such as satellite health status, through navigation messages. However, this information is delayed and is not delivered fast enough for applications that require high levels of safety. As it typically takes from 15 min to several hours to deliver warning to users this delay is problematic for applications that require high levels of safety and therefore these messages have no meaningful capabilities for integrity monitoring [6]. Instead, users can determine the integrity using either receiver autonomous integrity monitors (RAIMs), which have been used for many years in various application fields, or an external integrity data source from a GNSS augmentation system, such as ground-based augmentation systems (GBAS) or satellite-based augmentation systems (SBAS), to verify the integrity of the estimated position using reference receivers [7,8,9,10]. The basic concept of RAIM is to use redundant observations to identify and exclude erroneous measurements to ensure that the quality of the estimated position is reliable. The consistency of redundant measurements provides a clue as to whether the navigation satellites are operating out of their normal range and whether the position calculated with this error is unusable.

However, most studies related to RAIM have primarily focused on the flight phase of the non-precision approach (NPA), which can become an issue that needs to be addressed when RAIM is used for certain services, such as approach with vertical guidance (APV); the alert limits for NPA are quite moderate, but they are considerably tighter for the APV service. The APV service has lower alert limits because hazardously misleading information (HMI) events can occur when the position error exceeds the alert limit while the system is declared available [8,10,11,12,13]. Typical RAIMs cannot prove that an HMI event will be identified all the time with an overly high probability of ensuring integrity requirements. In addition, recent studies have focused more on improving the availability of RAIM in multi-constellation GNSS. This is being developed with dramatic progress and better performance, rather than on improving the RAIM integrity level [14,15,16,17]. The operation of autonomous vehicles for the implementation of ITS does not require a large separation distance to compensate for the limitations of navigation accuracy, which greatly improves the safety ratings. The extensive use of these autonomous GNSS-based techniques is currently constrained by strict safety regulations, such as the required navigation performance (RNP), which is specified in terms of accuracy and integrity requirements for a given operating mode. RNP was originally developed for air navigation and has since been extended to ground vehicle navigation [3,4]. Therefore, an alternative integrity augmentation mechanism is needed to detect error sources that exceed a predefined stringent safety limit and safely send alerts to manned/unmanned ground systems to avoid the constraints.

For this reason, the goal of this paper is to devise a new method to more efficiently capture anomalous behavior in a ranging signal. This method should have the reduced anomalies detection time in GNSS augmentation systems, while also increasing the availability of RAIM to meet the more strictly regulated service level. One possible way to more easily distinguish anomalies in observed GNSS signals is to take advantage of a time-delayed neural network (TDNN), which is a nonlinear model, as opposed to simply using RAIM alone. TDNNs can be used to estimate useful information about faulty characteristics, which are expressed in irregular sequences as the range residual calculated through received GNSS signals, by modeling the test statistic calculated over the existing RAIM. The association of the time series of the test statistic achieved from RAIM combined with TDNN for GNSS augmentation systems ultimately enables the computation of the dissimilarity between the current trend (based on online measurements) and the past trend (when the navigation satellites are under normal conditions) in order to increase navigation system integrity.

Two case studies have been investigated to assess the performance of the proposed integrity monitoring strategy for anomalous event detection in GNSS. One study uses data generated via software simulation and the other uses real GPS data collected from an integrity monitoring system installed on the rooftop of a multi-media building in Soonchunhyang University, Korea. During testing, we accounted for a step-type anomalous failure event, which results in an instantaneous large measurement jump. The test results demonstrate the feasibility of detecting a bad navigation satellite faster than typical RAIMs. This means the proposed method can be used with APV or intelligent transportation systems, which require much higher integrity conditions for safety, which are not achievable through RAIM. Employing TDNN-based integrity monitoring using RAIM is a very effective alternative to improve the assurance level of the integrity of typical RAIMs. The ability to detect anomalies has been improved, especially when there is an abnormality in the measurements, and our proposed method shows a clear advantage in being able to detect small variations, as compared to a typical monitor.

## 2. GNSS Signal Modeling

Supposing $\rho \in {\mathbb{R}}^{n\times 1}$ is the measurement of the pseudo-range obtained from navigation messages of *n* visible navigation satellites, the basic measurement formulation between the position of a user followed by a linearization and the difference of the predicted and observed measurements can be described by the following linear equation [6]:

(1) $$\delta y=H\delta x+\varepsilon ,\varepsilon \sim N\left(0,\sigma^{2}I\right)$$

Here, $\delta y\in {\mathbb{R}}^{n\times 1}$ is the measurement residual formed by the differences between the predicted range and the measured range on a basis of the nominal user position and the clock bias. Additionally, $H\in {\mathbb{R}}^{n\times 4}$ is the observation matrix formulated by linearization for the nominal user position and clock bias, while $\delta x\in {\mathbb{R}}^{n\times 1}$ is the navigation error state that includes three components of the actual position deviation from the nominal position plus the user clock bias deviation. Finally, $\varepsilon \in {\mathbb{R}}^{n\times 1}$ is the measurement error produced by the typical receiver noise, imprecise information of the satellite position and satellite clock error, unanticipated errors caused by satellite failure, and so on. These errors can take the form of a standard Gaussian distribution with zero mean and covariance $\sigma^{2}I$ [11,18]. Based on the fact that at least four satellites can be seen in most cases, redundant pseudo-range information is available, and the position estimation is attained from the best fit of the over-determined data. The least squares of the state at an instance *k* are estimated as follows:

(2) $$\delta x_{k,LS}=H_{k}^{+}\delta y_{k}$$

Here, $H_{k}^{+}$ denotes the pseudo inverse of $H_{k}$ (i.e., $H_{k}^{+}={\left(H_{k}^{T}H_{k}\right)}^{-1}H^{T}$), the estimate of $\delta y_{k}$ is $\delta \tilde{y_{k}}=H_{k}\delta x_{k,LS}$, and the error of the range residual is defined as $\delta \epsilon_{k}=\delta y_{k}-\delta \tilde{y_{k}}$. RAIM is the easiest and most cost-effective architecture for integrity monitoring, quantifying a self-consistency checking concept of redundant measurements. Its test statistic is described based on the sum of the squared range residual error as:

(3) $$\Xi_{RAIM,k}=\sqrt{\delta \epsilon_{k}{}^{T}\delta \epsilon_{k}/\sigma^{2}\left(n_{k}-4\right)}$$

The general threshold value of RAIM (i.e., $T_{RAIM,k}$) is directly computed by the cumulative distribution function of $\chi^{2}\left(n_{k}-4\right)$ at an instance *k* with desired values of $P_{FA}$ and $P_{MD}$: $T_{RAIM,k}=Q^{-1}(P_{FA}|n_{k}-4)$ [1,2]. Here, since the range residual error statistic is a quadratic form of $\delta \epsilon_{k}$, the probability of a false alarm (i.e., $P_{FA}$) is presented by the chi-squared distribution and is proportional to the normal random measurement noise $\varepsilon_{k}$. Once a satellite that has been corrupted by anomalies is noticed, it should be eliminated to ensure uninterrupted navigation.

## 3. TDNN for Anomalous Event Detection

Many applications require temporal information to be processed, such as trends in specific time windows, time series to a certain time, and so on. Common tasks dealing with temporal information include time-series prediction, classification, or mapping to other time series [19]. Long-term behavior can typically be modeled using neural networks (NNs) that have been successfully applied to compensate for the time delay in nonlinear processes. NNs may adopt two types of mechanisms (i.e., a non-recurrent structure based on delay elements in the feedforward direction and a recurrent structure with delayed feedback) to store temporal information internally. Unlike biological neural networks, connections between artificial neurons are not usually added or removed after the network has been created. Instead, connections are weighted and the weights are adapted by a learning algorithm. An input signal propagates through the network in the direction of the connections until it reaches the output of the network. In supervised learning, the learning algorithm adjusts the weights to minimize the discrepancy between the output of the network and the desired value provided [20,21,22].

The Recurrent Neural Network (RNN) architecture, e.g., Long Short Term Memory (LSTM), has been shown to effectively learn the temporal dynamics of the signal, but the training is complicated by the increased network complexity [23,24,25,26]. Another weakness of RNN is that the deeper the structure, the more difficult it becomes to learn as the gradient has to pass through several hidden states and be involved in optimization. When compared to recurrent structures in this aspect, feedforward learning is relatively fast, simple, and more efficient for modeling uncomplicated temporal dynamics. TDNNs, which were originally designed for speech recognition, perform exceptionally well when modeling dynamic systems with large time delays [27]. They are modified feedforward networks designed to capture the dynamics of modeled processes. Because a feedforward network has no internal memory to store information about the past, it is insufficient for processing temporal sequences [20]. To overcome this limitation, the memory of the past is introduced by means of extending the input of the network using tapped delay lines as synapses.

**Figure 1:**
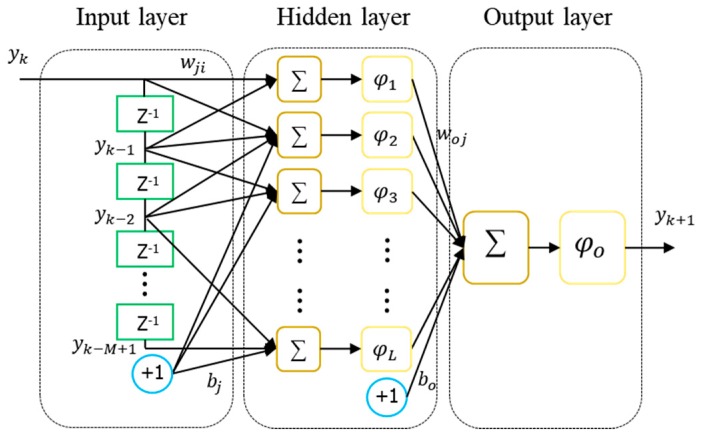
Architecture of the time-delayed neural network (TDNN).

For this reason, although there are various neural network structures for learning the long temporal dependencies, we adopted a TDNN to capture the GNSS anomalous behavior through the change of metric, which is less dynamically changed, and will be described later. Because there is no modification to the network topology, a standard learning technique is typically based on temporal backpropagation, and the weights that look at the gradient of the error surface in TDNNs are adjusted. The stimulus signal $y_{k}$ is captured and processed with synaptic weights w by the sum function, and the newly generated activation potential of the neuron is achieved by the activation function φ, as shown in Figure 1. Then, the output of the network is given by:

(4) $$y_{k+1}=\varphi_{o}\left(\sum_{j=1}^{L}w_{oj}\cdot \varphi_{j}\left(\sum_{i=1}^{M}y_{k-i+1}w_{ji}+b_{j}\right)+b_{o}\right)$$

Here, M is the number of inputs and L is the number of neurons in the hidden layer. The usual choice of an activation function is a sigmoid, $\varphi \left(t\right)=1/\left(1+e^{-t}\right)$, or hyperbolic tangent, $\varphi \left(t\right)=\left(e^{2t}-1\right)/\left(e^{2t}+1\right)$, but any differentiable function can be employed. The advantage of these two functions is that each first derivative can be represented by its own simple function.

Among the many algorithms used for training TDNNs, the Levenberg–Marquadt optimization scheme is considered to be the most effective [23,27,28,29,30]. The Levenberg–Marquadt method adaptively varies the parameter updates to reduce the sum of the squares of errors of the form:

(5) $$E\left(w\right)=\bar{e}{\left(w\right)}^{T}\bar{e}\left(w\right)=\overset{\infty }{\underset{k=0}{\sum }}\|e_{k}{\left(w\right)\|}^{2}$$

Here, $e_{k}\left(w\right)$ is the error in the *k*-th epoch and $\bar{e}\left(w\right)$ is a vector with element $e_{k}\left(w\right)$. If the difference between the former weight vector and the newly calculated weight vector is small, the error vector can be expanded into a first order through a Taylor series [31,32].

If a function E(w) needs to be minimized with respect to the weight vector w, then Newton’s method becomes:

(6) $$\Delta w=-{\left[\nabla^{2}E\left(w\right)\right]}^{-1}\nabla E\left(w\right)$$

Here, $\nabla^{2}E\left(w\right)$ is the Hessian matrix and ∇E(w) is the gradient. It can then be shown that:

(7) $$\nabla E\left(w\right)=J^{T}\left(w\right)\bar{e}\left(w\right)$$

(8) $$\nabla^{2}E\left(w\right)=J^{T}\left(w\right)J\left(w\right)+\sum_{k=1}^{N}e_{k}\left(w\right)\frac{\partial^{2}e_{k}\left(w\right)}{\partial w_{i}\partial w_{j}}$$

Here, J(w) is the Jacobian matrix, which consists of the first derivatives of the network errors regarding the weights and biases. It can be assumed that the second term of (8) is trivial, as compared to the product of the Jacobian matrix for the Gauss–Newton method, such that:

(9) $$\Delta w=-{\left[J^{T}\left(w\right)J\left(w\right)\right]}^{-1}J^{T}\left(w\right)\bar{e}\left(w\right)$$

In order to avoid the instance where the simplified Hessian matrix shown above is not invertible, the Levenberg–Marquadt modification of the Gauss–Newton method can be obtained as [31]:

(10) $$\Delta w=-{\left[J^{T}\left(w\right)J\left(w\right)+\gamma I\right]}^{-1}J^{T}\left(w\right)\bar{e}\left(w\right)$$

Here, I is the identity matrix and γ is a parameter used to ensure that the matrix $\left[J^{T}\left(w\right)J\left(w\right)+\gamma I\right]$ is positive definite, thereby making it invertible. Choosing a pertinent value of γ is extremely important for the function of the algorithm because it is responsible for the stability and convergence speed.

To provide information about GNSS anomalous events to users, we propose installing an additional integrity monitor on the augmentation system by identifying the observation of an irregular time series in the test statistic in terms of the range residual error along with an appropriate model. Naive approaches of modeling processes include methods like averaging past data points, returning a previous data point, and linear extrapolation. While these methods may be sufficient for some relatively simple time series, more sophisticated methods are required to cope with real-world time series, such as navigation signals [33,34].

TDNNs use historical data to estimate future values by finding a model of the process that empirically fits past data. Additionally, TDNNs that have noise suppression capabilities are referred to as finite impulse response (FIR) networks because of their tapped delay lines, which represent past activities. The irregularities observed in navigation measurements, which represent the intrinsic dynamics of the navigation satellites and/or the nonlinearity of the environments in the navigation signal path, can be captured by mapping the observable past terms to future ones [19]. TDNNs can later be united with the time series of the test statistic through typical RAIMs.

**Figure 2:**
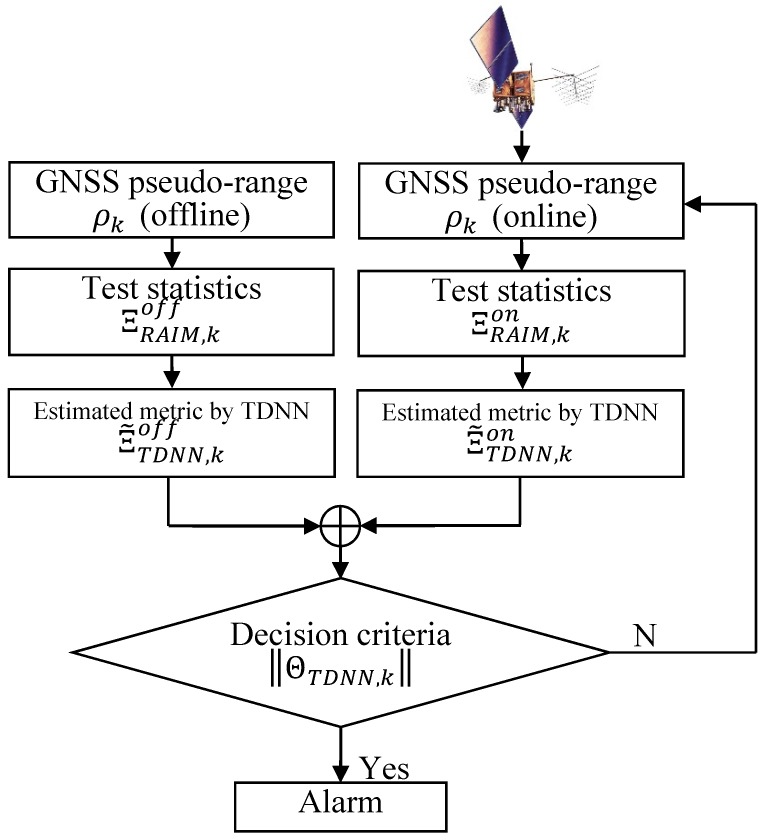
Schematic of the proposed integrity monitoring strategy based on estimated measurements made by TDNN.

Figure 2 depicts the overall schematic of the proposed approach for integrity monitoring. To ensure that the received navigation signal dynamics (which is the time series of the test statistic $\Xi_{RAIM,k}$) remain in the network input, the metric (which is achieved based on the sum of the squared range residual error calculated by a standard RAIM) is changed into the delayed form of the metric in time:

(11) $${\tilde{\Xi }}_{TDNN,k}=f\left(\Xi_{RAIM,k},\Xi_{RAIM,k-1},\ldots ,\Xi_{RAIM,k-M+1}\right)$$

Here, ${\tilde{\Xi }}_{TDNN,k}$ is the network output, which is the estimated quantity of $\Xi_{RAIM,k+1}$, and M is the number of tapped delay lines. Once the TDNN is trained, the newly proposed test statistic is created, as follows:

(12) $$\Theta_{TDNN,k}=\|{\tilde{\Xi }}_{TDNN,k}^{on}-{\tilde{\Xi }}_{TDNN,k}^{off}\|$$

Here, ${\tilde{\Xi }}_{TDNN,k}^{off}$ is the estimated output through the TDNN whose propagated input signal is formed on the basis of the offline pseudo-range obtained when GNSS satellites are under normal conditions. Alternatively, ${\tilde{\Xi }}_{TDNN,k}^{on}$ is the output estimated with the online pseudo-range. This approach responds directly to the dissimilarity between ${\tilde{\Xi }}_{TDNN,k}^{off}$ (representing an ordinary state) and ${\tilde{\Xi }}_{TDNN,k}^{on}$ when the output is contaminated by anomalies. Any peak in the computed discrepancy between these is used to trigger an alarm. This enhanced inconsistency activates a caution message.

The detection threshold is generally computed with the mean and standard deviation of the metric $\Theta_{TDNN}$, which is obtained in (12):

(13) $$T_{TDNN}=\mu \left(\Theta_{TDNN}\right)\pm K_{ffd}\cdot INF\cdot \sigma \left(\Theta_{TDNN}\right)$$

Here, $\mu \left(\Theta_{TDNN}\right)$ and $\sigma \left(\Theta_{TDNN}\right)$ are the mean and standard deviation of $\Theta_{TDNN}$, respectively. $K_{ffd}$ is a multiplier used to satisfy the probability of fault-free detection given by the requirement conditions; for code-carrier divergence monitors supporting the use of ground-based augmentation systems (GBAS) in CAT-I operation this specific value is $1\times 10^{-8}$. An inflation factor INF, which is an inflated value of the standard deviation, overbounds the true error distribution to avoid continuity risks [34,35].

In summary, the role of the test statistic in (13) is to measure any differences between the estimated quantities of ${\tilde{\Xi }}_{TDNN,k}^{off}$ and ${\tilde{\Xi }}_{TDNN,k}^{on}$. This measurement changes the consistency of the estimates based on assumptions related to GNSS anomalies. Then, the test statistic in (13) is compared with the thresholds, which are pre-specified as described in (3), to see if an alarm should be published.

## 4. Simulation Case Studies

**Figure 3:**
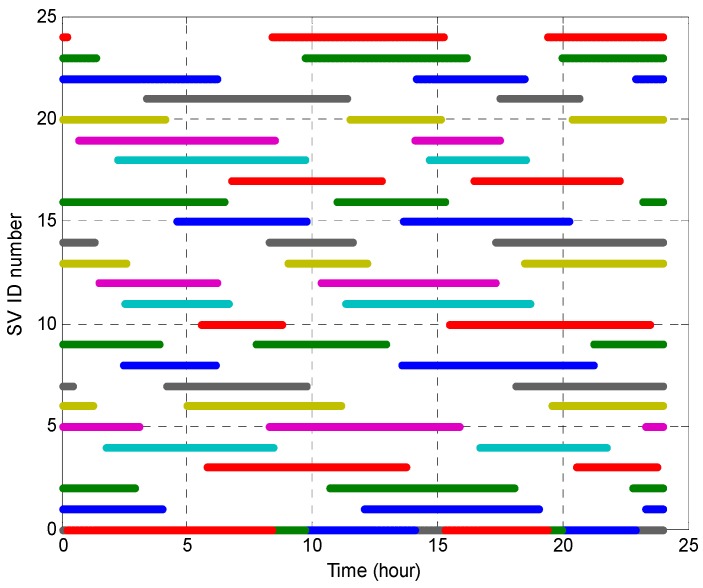
Exposed time of GPS satellites visible when the elevation mask is five degrees.

In this section, we consider two case studies to evaluate the capabilities of the proposed integrity monitoring architecture for anomalous event detection in GNSS. One case study uses synthetic data generated by software simulation and the other uses raw GPS data saved from an installed integrity monitor. For testing and evaluation purposes, we first simulated a 24-h-long GPS data sample using a virtual reference station (36.76907° N, 126.93490° E) located on the rooftop of a multi-media building in Soonchunhyang University, Korea. Figure 3 shows the exposed time of the GPS satellites seen through the synthetic data with an elevation mask set to five degrees; this shows that the number of visible satellites is at least nine.

The aim of the case study with the synthetic data is to show that the proposed monitoring for augmentation systems can shorten the detection time of an anomalous event, allowing this method to be used in safety and critical transportation applications. The test statistic of the standard RAIM architecture (i.e., $\Xi_{RAIM,k}$) in (3) was initially obtained through the received GPS signals and used to detect any divergence between the normal and abnormal states. Subsequently, the obtained metric was fed into the TDNN to be trained based on the Levenberg–Marquadt algorithm derived in (10) such that the TDNN showed the relationship of (11). Next, the estimated quantity of $\Xi_{RAIM,k+1}$ (i.e., ${\tilde{\Xi }}_{TDNN,k}$) obtained through the trained TDNN was used to form the newly proposed test statistic by comparing estimates based on the offline pseudo-range (extracted when the navigation messages are healthy) and estimates using the online pseudo-range.

**Figure 4:**
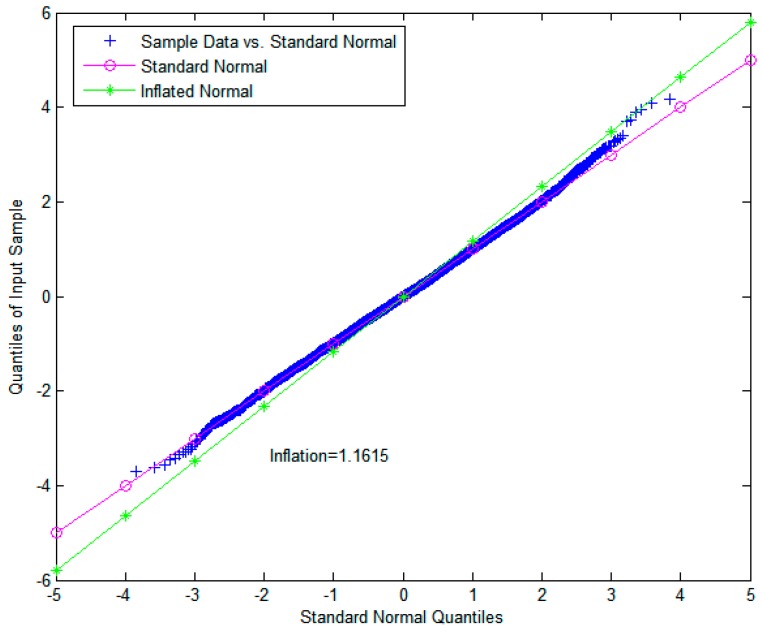
QQ plot for determination of the inflation factor using GPS synthetic data.

**Figure 5:**
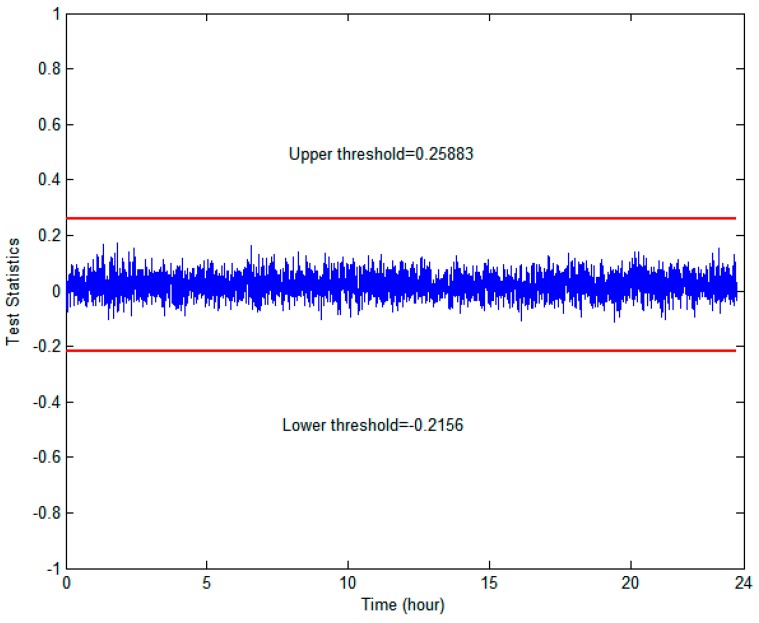
Proposed test statistics and resultant thresholds.

The proposed monitor $\Theta_{TDNN}$, represented by RAIM/TDNN, was evaluated against a standard integrity monitor $\Xi_{RAIM}$, represented by RAIM. The threshold value of RAIM (i.e., $T_{RAIM}$) was directly computed by the cumulative distribution function of $\chi^{2}\left(n_{k}-4\right)$ to satisfy the required probability of a false alarm (i.e., $1\times 10^{-8}$) to support the use of GBAS in CAT-I operation [35]. Additionally, the detection threshold of $\Theta_{TDNN}$ (i.e., $T_{TDNN}$ in (13)) was computed based on the mean and standard deviation of $\Theta_{TDNN}.$ Finally, $K_{ffd}$ was selected to be 5.73 to satisfy the probability of fault-free detection specified by the continuity requirement of $1\times 10^{-8}$, and the original standard deviation was inflated by the inflation factor *INF* = 1.1615, which overbounds the true error distribution to avoid continuity risks, as shown in Figure 4. In Figure 5, the metric of RAIM/TDNN and its determined upper and lower thresholds are shown.

**Figure 6:**
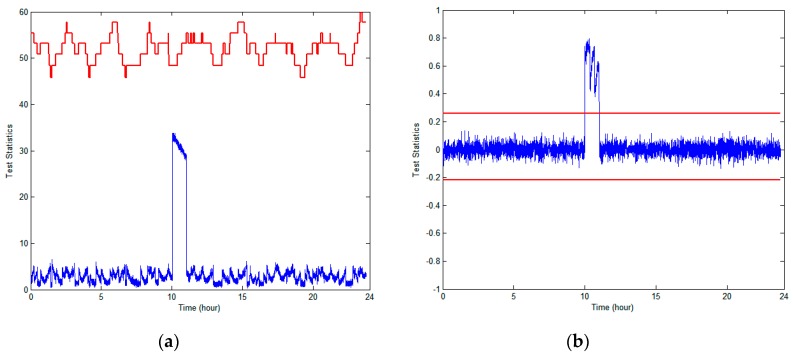
Performance comparison of anomaly detection schemes when a +100-m bias was injected into the observed PRN9 from the GPS synthetic data: (**a**) receiver autonomous integrity monitor (RAIM) and (**b**) RAIM/TDNN.

For the tests, we took into account a step-type anomalous failure event, which results in an instantaneous large measurement jump, such as a sudden change in the GPS receiver clock bias due to an oscillator or an erroneous upload of the ephemeris. To simulate the effects of this instantaneous jump error, we first inserted a 100-m bias into the pseudo-range measurement of PRN9 at 10 a.m. for an hour; it seems that there is no possibility of reliable detection of such defects by RAIM, as shown in Figure 6. In contrast, we observed that RAIM/TDNN can perfectly sense this type of jump error; thus, the capability of failure detection of RAIM/TDNN is far superior to that of RAIM.

**Figure 7:**
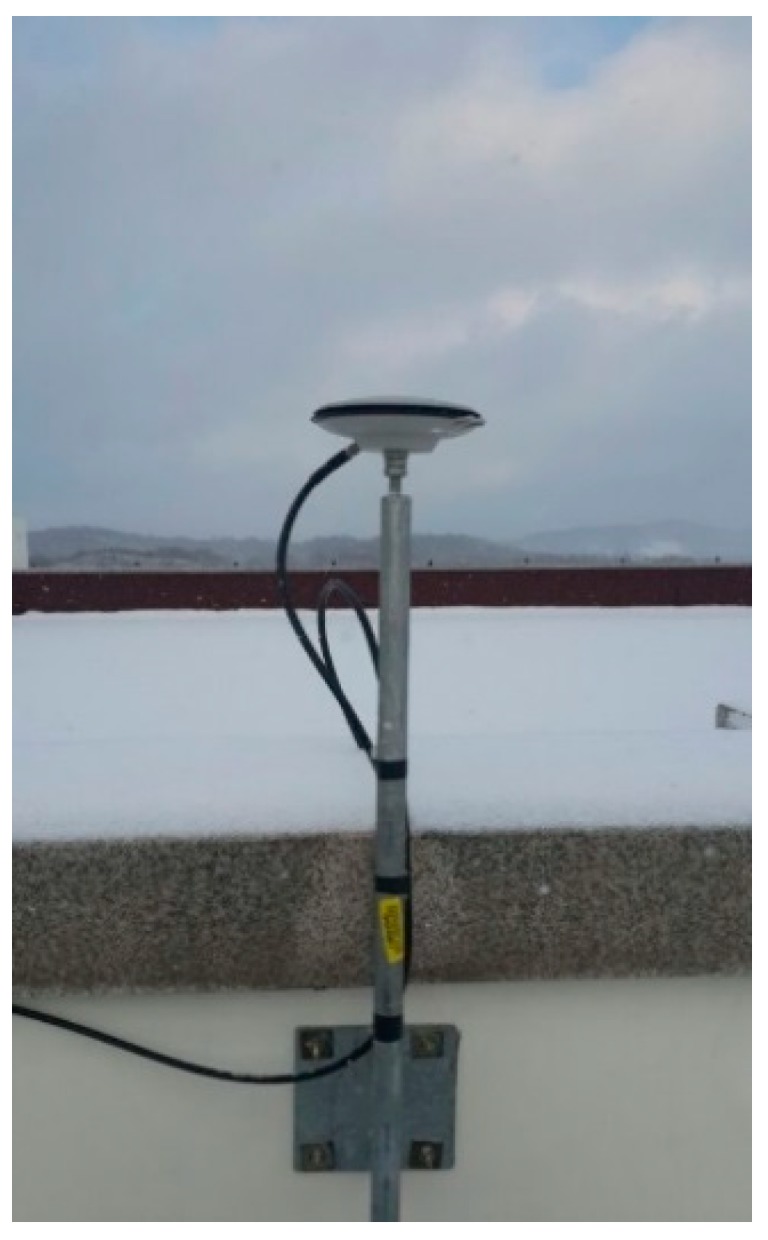
GPS antenna mounted on the side wall of a roof.

**Figure 8:**
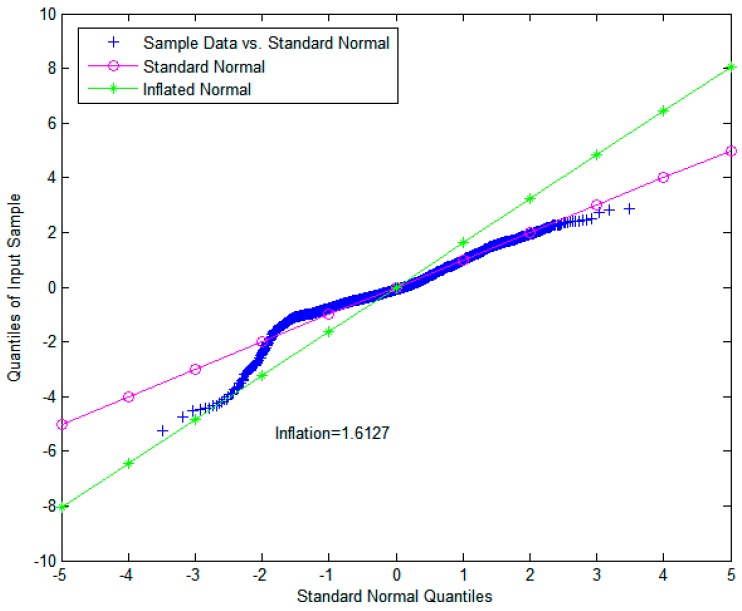
QQ plot for determination of the inflation factor using GPS raw data.

**Figure 9:**
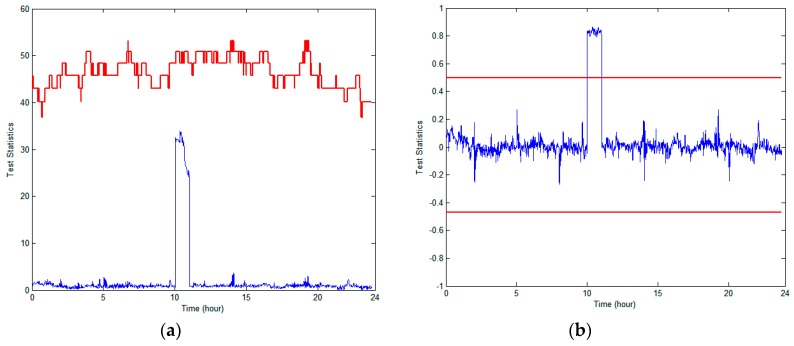
Performance comparison of anomaly detection schemes when a +100-m bias was injected into the observed PRN9 from the GPS raw data: (**a**) RAIM and (**b**) RAIM/TDNN.

For an additional assessment of the proposed RAIM/TDNN, raw data were obtained from a GPS antenna-receiver module, which consisted of a NovAtel GPS-703-GGG antenna and a NovAtel Flexpak6 receiver. The GPS antenna was installed on the rooftop ($36.76907^{{}^{\circ}}\;N,\;126.93490^{{}^{\circ}}\;E$) of a multi-media building in Soonchunhyang University, Korea, as shown in Figure 7. The receiver was connected directly to a PC that continuously stores GPS raw data using a 1-Hz sampling rate. The proposed RAIM/TDNN architecture was tested for 24 h periods and data sets were archived for 3 July, 6 July, and 7 July 2018. The detection threshold of RAIM/TDNN (i.e., $T_{TDNN}$) was computed using the mean and standard deviation of $\Theta_{TDNN}$. Additionally, $K_{ffd}$ and *INF* were selected to be 5.73 and 1.6127, respectively, to satisfy the probability of fault-free detection specified by the continuity requirement of $1\times 10^{-8}$, as shown in Figure 8. For the tests, the pseudo-range measurements of PRN9 were contaminated by adding a constant bias error (+100 m) for an hour at 10 am, without loss of generality. As shown in Figure 9, which is analogous to the previous test results using synthetic data, the proposed RAIM/TDNN detected this error level well, whereas the existing RAIM did not detect it at all. It should be noted that such an error level, which may be significant for reliable and seamless navigation, can be ignored without quickly responding and issuing an alarm with RAIM.

**Figure 10:**
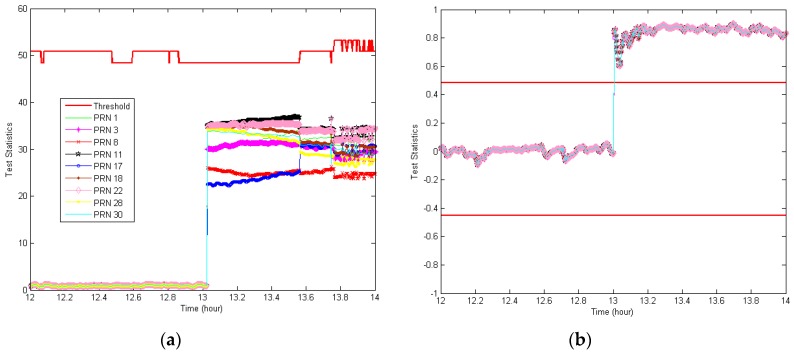
Performance comparison of anomaly detection schemes when a +100-m bias was alternately applied to all visible satellites from 1 p.m. to 2 p.m.: (**a**) RAIM and (**b**) RAIM/TDNN.

To further assess the proposed RAIM/TDNN monitor, we applied a +100-m bias alternately to all visible satellites over a period of one hour starting at 1 pm. The results, shown in Figure 10, once again confirm that the proposed anomaly detection scheme is capable of detecting bad navigation satellites far faster and more reliably than RAIM for all visible satellites. To evaluate the generalized performance, the anomaly detection performance of RAIM/TDNN was tested in terms of the detection rate and compared with that of RAIM when injecting a +150-m bias for an hour while changing the injection start time for all active satellites. These results showed that the proposed RAIM/TDNN monitor is considerably more suitable for detecting abnormalities than currently used RAIM, as shown in Table 1. RAIM exhibited instability with wide detection rates ranging from 0% to 99.7%, whereas RAIM/TDNN consistently showed detection rates of 100%. It was confirmed, once again, that the proposed RAIM/TDNN is more likely to detect faulty measurements.

## 5. Conclusions

The reliability of a navigation system is a very important factor in areas where strict performance is required (e.g., civil aviation and ITS). Therefore, integrity monitoring is an inseparable part of safety-critical navigation applications. To ensure system integrity, a navigation system should quickly detect faults or failures and promptly warn users. Although a standard RAIM technique has been devoted to GNSS to provide integrity monitoring within avionics itself, such as in civil aviation for LNAV or NPA, typical RAIMs cannot guarantee that an HMI event will be identified all the time with an overly high probability of ensuring tighter integrity requirements. In addition, RAIM is insufficient for on-ground vehicle integrity monitoring of various types of location-based ITS applications, including vehicles that carry hazardous materials and GNSS-based electronic fee collection.

To overcome such limitations of RAIM, we proposed an efficient way to ensure more stringent integrity requirements by capturing unusual behavior through GNSS augmentation systems while significantly reducing the anomalies detection time. A TDNN incorporated within RAIM was used to more clearly distinguish anomalies in observed GNSS signals to estimate useful information about the faulty characteristics, rather than simply using RAIM alone. By combining the test statistics from RAIM and a TDNN, we can compute the dissimilarity between the current trend (based on online measurements) and the past trend (where GNSS satellites were in a normal state). This allows the integrity of the navigation system to be enhanced. Performance assessments of the proposed integrity monitor were conducted based on raw GPS data and data sets generated by software simulation. Based on this performance evaluation, it was confirmed that the defects of navigation satellites can be detected more reliably and quickly using our proposed system, as compared with conventional RAIM. Abnormal phenomena at a level that could not be sensed via RAIM were detected, thus improving the ability to detect abnormalities.

Based on these results, GNSS augmentation systems can be easily constructed by using the proposed enhanced integrity monitor. Therefore it is applicable to ITS fields sensitive to the reliability of location information. It is also expected that it can serve as another integrity monitor for improving performance of GBAS or SBAS in the aviation sector. It is necessary to conduct an analysis of whether the proposed monitor can be installed in the GBAS ground facility for use as a CAT-II/III monitor in the future.

## Figures and Tables

**Table 1:** Detection rate when injecting a +150-m bias for an hour while changing the injection start time for all active satellites.

PRN	Block-Type	Bias Injection Start Time (h)	Detection Rate (%)		PRN	Block-Type	Bias Injection Start Time (h)	Detection Rate (%)	
			RAIM	RAIM/TDNN				RAIM	RAIM/TDNN
1	IIF	11	13.6	100	17	IIR-M	13	0.3	100
2	IIR	19	55.8	100	18	IIA	10	47.8	100
3	IIF	6	48.3	100	19	IIR	14	1.67	100
4	N/A	-	-	-	20	IIR	23	99.7	100
5	IIR-M	19	55.8	100	21	IIR	7	63.9	100
6	IIF	14	4.4	100	22	IIR	6	41.6	100
7	IIR-M	9	0.8	100	23	IIR	6	0.3	100
8	IIF	8	99.7	100	24	IIF	23	98.6	100
9	IIF	8	15.0	100	25	IIF	5	34.2	100
10	IIF	11	0.0	100	26	IIF	5	97.5	100
11	IIR	10	47.8	100	27	IIF	11	33.6	100
12	IIR-M	16	50.3	100	28	IIR	12	16.7	100
13	IIR	19	53.1	100	29	IIR-M	5	71.1	100
14	IIR	6	66.9	100	30	IIF	11	20.3	100
15	IIR-M	21	99.7	100	31	IIR-M	7	99.7	100
16	IIR	11	13.1	100	32	IIF	5	70.0	100

## References

[1] Parkinson B.W., Axelrad P. (1988). Autonomous GPS integrity monitoring using the pseudorange residual. Navig. J. Inst. Navig..

[2] Sturza M.A. (1988). Navigation system integrity monitoring using redundant measurements. Navig. J. Inst. Navig..

[3] Velaga N.R., Quddus M.A., Bristow A.L., Zheng Y. (2012). Map-aided integrity monitoring of a land vehicle navigation system. IEEE Trans. Intell. Transp. Syst..

[4] Binjammaz T.A., Al-Bayatti A.H., Al-Hargan A.H. (2016). Context-aware GPS integrity monitoring for intelligent transport systems. J. Traffic Transp. Eng..

[5] Brown R.G., Hwang P. (1986). GPS failure detection by autonomous means within the cockpit. Navig. J. Inst. Navig..

[6] Misra P., Enge P. (2006). Global Position System: Signals, Measurements, and Performance.

[7] Yang L., Zhang Y., Gao Y. Enhanced RAIM based on weighted and subset schemes for GNSS receiver. Proceedings of the IEEE/ION PNT.

[8] Shi Y., Teng Y. (2012). The clock-aided RAIM method and its application in improving the positioning precision of GPS receiver. Acta Astronaut..

[9] Lee Y.C. (2013). New advanced RAIM with improved availability for detecting constellation-wide faults, using two independent constellations. Navig. J. Inst. Navig..

[10] Fu L., Zhang J., Li R., Cao X., Wang J. (2015). Vision-aided RAIM: A new method for GPS integrity monitoring in approach and landing phase. Sensors.

[11] Rakipi A., Kamo B., Cakaj S., Kolici V., Lala A., Shinko I. (2015). Integrity monitoring in navigation systems: Fault detection and exclusion RAIM algorithm implementation. J. Comput. Commun..

[12] Borio D., Gioia C. (2016). Galileo: The added value for integrity in harsh environments. Sensors.

[13] Wang E., Jia C., Tong G., Qu P., Lan X., Pang T. (2018). Fault detection and isolation in GPS receiver autonomous integrity monitoring based on chaos particle swarm optimization-particle filter algorithm. Adv. Space Res..

[14] Martini I., Rippl M., Meurer M. Advanced RAIM Architecture Design and User Algorithm Performance in a Real GPS, GLONASS and Galileo Scenario. Proceedings of the IEEE/ION ITM.

[15] Belabbas B., Gass F. RAIM Algorithms Analysis for a Combined GPS/GALILEO Constellation. Proceedings of the IEEE/ION ITM.

[16] Zhang P., Chen P., Song D., Fan G. (2018). Research on GNSS Receiver Autonomous Integrity Monitoring Method Based on M-Estimation. Math. Probl. Eng..

[17] Meng F., Wang S., Zhu B. (2015). GNSS Reliability and Positioning Accuracy Enhancement Based on Fast Satellite Selection Algorithm and RAIM in Multiconstellation. IEEE A&E Syst. Mag..

[18] Cho J. (2016). On the enhanced detectability of GPS anomalous behavior with relative entropy. Acta Astronaut..

[19] Ren X.M., Rad A.B. (2007). Identification of nonlinear systems with unknown time delay based on time-delay neural networks. IEEE Trans. Neural Netw..

[20] Fuchs E., Gruber C., Reitmaier T., Sick B. (2009). Processing short-term and long-term information with a combination of polynomial approximation techniques and time-delay neural networks. IEEE Trans. Neural Netw..

[21] Meng H., Bianchi-Berthouze N., Deng Y., Cheng J., Cosmas J.P. (2016). Time-delay neural network for continuous emotional dimension prediction from facial expression sequences. IEEE Trans. Cybern..

[22] Wohler C., Anlauf J.K. (1999). A time delay neural network algorithm for estimating image-pattern shape and motion. Image Vis. Comput..

[23] Hochreiter S., Schmidhuber J. (1997). Long short-term memory. Neural Comput..

[24] Peddinti V., Povey D., Khudanpur S. A Time Delay Neural Network Architecture for Efficient Modeling of Long Temporal Contexts. Proceedings of the INTERSPEECH.

[25] Sak H., Senior A., Beaufays F. (2014). Long Short-Term Memory Based Recurrent Neural Network Architectures for Large Vocabulary Speech Recognition. arxiv.

[26] Graves A., Mohamed A., Hinton G. Speech Recognition with Deep Recurrent Neural Networks. Proceedings of the IEEE Acoustics, Speech and Signal Processing.

[27] Waibel A., Hanazawa T., Hinton G., Shikano K., Lang K. (1989). Phoneme recognition using time-delay neural networks. IEEE Trans. Acoust. Speech Signal Process..

[28] Charalambous C. (1992). Conjugate gradient algorithm for efficient training of artificial neural networks. IEE Proc..

[29] Battiti R. (1992). First- and Second-Order Methods for Learning: Between Steepest Descent and Newton’s Method. Neural Comput..

[30] Wilamowski B.M., Chen Y. Efficient Algorithm for Training Neural Networks with one Hidden Layer. Proceedings of the IJCNN.

[31] Hagan M.T., Menhaj M.B. (1994). Training feed forward network with the Marquardt algorithm. IEEE Trans. Neural Netw..

[32] Chen T.C., Han D.J., Au F.T.K., Than L.G. (2003). Acceleration of Levenberg-Marquadt training of neural networks with variable decay rate. IEEE Trans. Neural Netw..

[33] Cho J., Yun Y., Heo M. (2015). GBAS ionospheric anomaly monitoring strategy using Kullback-Leibler divergence metric. IEEE Trans. Aerosp. Electron. Syst..

[34] Yun Y., Cho J., Heo M. Automated determination of fault detection thresholds for integrity monitoring algorithms of GNSS augmentation systems. Proceedings of the IEEE/ION PLANS.

[35] Radio Technical Commission for Aeronautics (RTCA) (2006). Minimum Operational Performance Standards for Global Positioning System/Wide Area Augmentation System Airborne Equipment.

